# Parenting Interventions to Prevent and Reduce Physical Punishment: A Scoping Review †

**DOI:** 10.3390/ijerph21111539

**Published:** 2024-11-20

**Authors:** Isabel Garces-Davila, Ashley Stewart-Tufescu, Janice Linton, Julie-Anne McCarthy, Sonya Gill, Aleksandra Ciochon Newton, Samantha Salmon, Tamara Taillieu, Tracie O. Afifi

**Affiliations:** 1 Department of Community Health Sciences, University of Manitoba, Winnipeg, MB R3E 0W3, Canada; ummcca26@myumanitoba.ca (J.-A.M.); gills348@myumanitoba.ca (S.G.); samantha.salmon@umanitoba.ca (S.S.); tamara.taillieu@umanitoba.ca (T.T.); tracie.afifi@umanitoba.ca (T.O.A.); 2 Faculty of Social Work, University of Manitoba, Winnipeg, MB R3T 2N2, Canada; ashley.stewart-tufescu@umanitoba.ca; 3 Children’s Hospital Research Institute of Manitoba, University of Manitoba, Winnipeg, MB R3T 2N2, Canada; 4 Neil John Maclean Health Sciences Library, Bannatyne Campus, University of Manitoba, Winnipeg, MB R3E 0W3, Canada; janice.linton@umanitoba.ca; 5 Department of Statistics, University of Manitoba, Winnipeg, MB R3T 2N2, Canada; 6 Departments of Psychiatry, University of Manitoba, Winnipeg, MB R3T 2N2, Canada

**Keywords:** parenting interventions, physical punishment, scoping review

## Abstract

Physical punishment is the most common form of violence against children worldwide and is associated with an increased risk of long-term adverse outcomes. Interventions targeting parents/caregivers are frequently implemented to prevent and reduce the use of physical punishment. This scoping review aimed to map the existing literature on evidence-informed parenting interventions targeting physical punishment. A scoping review following the World Health Organization (WHO) Review Guide, the Joanna Briggs Institute (JBI) 2020 Guide for scoping reviews, was conducted to address the objective of this review. An academic health sciences librarian systematically searched electronic databases (EBSCO, MEDLINE, EMBASE, SCOPUS) for peer-reviewed journal articles. Two reviewers independently screened titles and abstracts, followed by a full-text review according to inclusion and exclusion criteria following the Participants, Concept, and Context framework. Eighty-one studies were included for full-text eligibility. The results suggest that most interventions examined were conducted in North America, targeted mothers and fathers, and were delivered in person. The results from this scoping review describe the state of evidence-informed parenting interventions to prevent and reduce physical punishment. This review found opportunities for future research to implement effective parenting interventions on a larger societal scale and use mixed methods approaches to evaluate parenting interventions.

## 1. Introduction

Two out of three children aged 1 to 14 years are exposed to physical punishment at home [1], and annually, 80% of children experience some form of physical punishment (e.g., spanking, hitting or slapping on the hand, arm, or leg) from their parents [2]. In addition to prevalence studies, a growing body of literature consistently demonstrates that physical punishment in childhood is associated with an increased risk of long-term detrimental cognitive [3,4,5,6,7], behavioural [3,4,6,8], physical [8], and mental health outcomes [6,7,9,10,11,12]; risk of experiencing other forms of physical violence [13,14]; and risk of violent behaviour spectrum (e.g., violent crime) [15]. Notably, the results of a 2022 rapid review of physical punishment and child development confirm previous studies [6,7]; there is no evidence that physical punishment is associated with any positive developmental, physical, or mental health outcomes for children and youth [4].

In response to this public health concern, the United Nations Convention on the Rights of the Child [16,17], the Sustainable Development Goals (SDGs) [18], professional healthcare organizations [19,20], and scholars [11,21,22] have advocated for the elimination of physical punishment, affirming that it violates children’s rights [22] and increases the risk of physical injury [13] and the involvement of child protection services [23].

### 1.1. Defining Physical Punishment

Definitions of physical punishment vary. For example, physical or corporal punishment, referred to as spanking, smacking, or slapping [10], is defined as ‘any punishment in which physical force is used and intended to cause some degree of pain or discomfort, however light [16]’. Another definition identifies physical punishment as using physical force to cause a child to experience pain without the intention to harm or to cause injury, to correct or control the child’s behaviour [5,24]. Of note, physical punishment and physical abuse are often considered distinct from adults’ perspectives [6,7,8]; however, both correspond to the continuum of parent–child violence [14,25], and their effects on development and well-being are similar [26,27]. There is a growing perspective that physical punishment could be considered a form of child maltreatment [11,28]. This view is supported by research indicating that various types of physical punishment are correlated with both physical and emotional abuse, suggesting that physical punishment has a similar underlying process to other forms of maltreatment [11,25]. Regardless of the caregiver’s intention or perspective, when a child is physically punished for modifying perceived misbehaviour, it causes physical and mental pain and distress, which has been noted as a similar physiological consequence of physical abuse [29]. Given the varying definitions of physical punishment, this scoping review considered studies that examined interventions to address parental use of physical and/or corporal punishment in the context of discipline or parent–child conflict. This definition allows for broad inclusion criteria of the multiple purposes of physical punishment presented in the literature.

Furthermore, recognizing the diverse terminology used in the literature to address physical punishment—such as corporal punishment, harsh parenting, physical discipline, smacking, and slapping—this study aimed to evaluate a wide range of research encompassing these concepts. By including studies that refer to both “corporal punishment” and “physical punishment”, along with other relevant terms, we hoped to provide a more comprehensive understanding of the topic. Please refer to Appendix A for a detailed list of the terms included in our search strategy.

### 1.2. Parenting Interventions to Address Child Maltreatment and Physical Punishment

#### 1.2.1. Parenting Interventions to Address Child Maltreatment

The topic of parenting strategies and interventions to address violence towards children, including various forms of child maltreatment, has been extensively explored in previous literature reviews. In 2023, the WHO conducted a series of reviews that focused on the effectiveness of parenting interventions and their societal implications [30]. The report was based on systematic reviews that examined broader questions regarding the acceptability, feasibility, balance of benefits and harms, and societal, economic, equity, and human rights implications of parenting interventions [30]. In line with this, Altafim and Linhares [31] conducted a systematic review that included parenting interventions to prevent violence, reduce child maltreatment, and promote positive parenting practices. Other systematic and scoping reviews with similar objectives can also be noted. For instance, Branco and colleagues [32] reported that parenting outcomes improved post-intervention, along with enhanced parental mental health.

Similarly, Casillas and colleagues [33] investigated home-based parenting programs and indicated that factors such as training, supervision, and fidelity monitoring had a significant impact on child maltreatment program outcomes. Additionally, Morello and colleagues [34] examined parenting interventions to reduce physical child maltreatment re-occurrence and reported a noteworthy reduction in recidivism rates and maltreatment risk, as well as improvements in parent–child relationships.

#### 1.2.2. Parenting Interventions to Address the Use of Physical Punishment

Previous reviews have explored parenting programs aiming to address child maltreatment and violence against children and youth, encompassing various forms of maltreatment. Physical punishment is acknowledged as a type of child maltreatment and is associated with both physical and emotional abuse [11,28]. However, this scoping review specifically focuses on parenting interventions aimed at reducing the use of physical punishment. The overwhelming evidence of the harms associated with physical punishment and the increasing global recognition of it as a violation of children’s rights have prompted calls to action from several international health and child-serving organizations to abolish the practice [35]. The INSPIRE framework, developed in collaboration with the WHO and numerous global child health and development organizations, including UNICEF, the Centres for Disease Control and Prevention (CDC), the Pan American Health Organization, and the World Bank Group, consists of seven strategies to reduce and prevent violence against children [35]. Strategy number 4: Parent and caregiver support calls for a reduction in harsh parenting and the promotion of positive child-rearing strategies [35]. Parenting interventions are cost-effective ways to strengthen the parent–child relationship and protect the child’s health, safety, and resilience, and they have long-lasting positive effects in preventing all types of violence throughout children’s lives from infancy into adulthood [35].

Parenting interventions to prevent and reduce physical punishment are often categorized as primary, secondary, or tertiary, serving universal, at-risk, or selective populations [36]. Universal populations include the general public [14]. At-risk populations include parents who have physically maltreated their children and are a target for intensive interventions aimed at preventing recurrence [14]. Selective populations include all parents and professionals who may influence parents’ decisions about discipline [37]. Primary interventions are universally implemented across all groups to address the risk factors of physical punishment before its occurrence [36]. Effective primary prevention approaches include media campaigns (Children See, Children Learn [22]) and group-based educational programs (Positive Discipline in Everyday Parenting [38]). Secondary-level interventions target selective populations, such as those at risk of engaging in physical child punishment [34,36]. Effective interventions for at-risk groups include the Nurse–Family Partnership home visitation program and the Incredible Years Parenting Training Program [36]. Tertiary-level programs aim to prevent physical punishment re-occurrence with parents previously identified as having maltreated their children or who have been referred for more individualized and specialized treatment [36]. Parent–Child Interaction Therapy (PCIT), Alternatives for Families: Cognitive Behavioral Therapy (AF-CBT), and Multisystemic Therapy (MST) are examples of interventions used to prevent physical punishment re-occurrence [39].

While the literature addressing parenting interventions has been represented by individual studies of parenting programs in which the primary outcomes reported are specific to the child-level outcomes (e.g., child compliance), in recent years, there has been a shift towards assessing interventions aimed at changing parent-level outcomes (e.g., parental use of physical punishment [18,30,40,41,42]). A qualitative review of parenting interventions to prevent re-occurrence found that effective interventions address parental self-management and provide alternative non-physical discipline strategies [43]. Although systematic and scoping reviews support parenting interventions (e.g., ACT Raising Safe Kids Parenting Program) to prevent and reduce physical punishment [26,37,44], research also shows inconclusive findings. For example, a review of randomized controlled trials of group-based and one-to-one parenting programs reported no evidence that parenting interventions prevented child physical punishment and neglect re-occurrence (e.g., no significant differences in the report of injuries in intervention and control groups) [45]. While such findings are informative, some limitations should be noted. This review only included studies based on randomized interventions, excluding mixed methods interventions or observational studies, and it lacked a definition of physical punishment in its inclusion and exclusion criteria [45].

The varying definitions of child physical punishment and physical abuse in parenting interventions have also been noted as a limitation in detecting and evaluating effective interventions [46,47]. Yoon and colleagues [47,48] conducted a systematic review to assess parent-reported child maltreatment measures’ sensitivity to detect change (before and after interventions). Among the 69 studies retrieved and 15 instruments of child maltreatment assessed (e.g., Conflict Tactics Scales—CTSPC, International Society for the Prevention of Child Abuse and Neglect Child Abuse Screening Tool for use in Trials—ICAST-Trial [46]), only the physical abuse subscale of the ICAST-Trial had high-quality evidence of responsiveness before and after interventions [47]. Further, the authors suggest that future research should account for the sensitivity to detect change in child maltreatment measures used to evaluate change after parents attend programs or interventions [47].

Another systematic review focused on positive discipline alternatives to physical punishment in schools and home settings [44]. The results from this review indicate that over 50 instruments (e.g., behaviour contracts, communication, modelling) used in parenting interventions show positive effects on child behaviour [44]. It is important to mention that this review is similar to others [18,49] that examined parent-reported changes in child behaviour as their primary outcome after intervention. The numerous studies and reviews in the interdisciplinary field covering the terms ‘discipline’ and ‘punishment’ are vast [44]. Some relevant studies might have been overlooked in a scoping review by Quail and Ward [44] due to the lack of specificity in defining physical punishment and related terms. Also, this review, similar to Gubbels and van der Put [49], included changes in child behaviour to assess the effectiveness of parenting interventions. However, the success of interventions to reduce parental use of physical punishment should rely on the assessment of parent-specific outcomes (e.g., attitudes towards physical punishment and use of punishment and non-violent disciplinary alternatives) and child and youth assessment of their parent’s behaviour as a result of the intervention [37].

Although findings from the various reviews mentioned offer important insights to further the investigation of parenting interventions to prevent and reduce physical punishment, critical literature gaps from previous studies should be noted, including: (a) most studies focus on parenting programs that primarily target child behaviour change [44,49] instead of assessing changes in parenting attitudes and behaviours related to physical punishment and positive discipline strategies and without the inclusion of children’s feedback on parental behaviour [50,51]. When children are part of interventions, this is measured via child reports of parenting behaviours after interventions; (b) the lack of a definition of physical punishment to guide inclusion and exclusion criteria is often not noted in most reviews [18,41,52]; (c) some interventions are not adequately adapted or transposed following cultural norms to certain low–middle-income countries (LMICs) [53]; and (d) given the numerous studies that use an experimental design to test parenting interventions, most reviews focus solely on randomized controlled trials (RCTs; e.g., [39]), overlooking those studies following quasi-experimental or observational designs. Examining RCTs alongside observational designs can provide relevant information on program components and the delivery of interventions.

### 1.3. Significance

This scoping review aimed to thoroughly examine the existing literature on parenting interventions that aim to reduce and prevent physical punishment against children and youth. This review followed a universal prevention approach with the ultimate goal of creating a culture of child safety. The aim was to present information that could be used to implement preventive programs, toward reducing physical punishment among all populations. This scoping review also offers information about programs promoting social norms and attitudes toward child well-being while engaging parents and offspring in ongoing efforts to protect children from the harmful impact of physical punishment. Our research question is formulated in general terms to offer a comprehensive overview of parenting intervention studies: “What is the evidence regarding parenting interventions to prevent and reduce physical punishment?” Our scoping review follows five objectives: (1) identifying evidence-informed parenting interventions to address physical punishment globally; (2) examining the results of parenting interventions; (3) assessing intervention delivery methods and considering children and youth’s perspectives; (4) emphasizing the measures used to evaluate the effectiveness of identified interventions; and (5) identifying facilitators and barriers to access, use, and implementation of interventions. We attempt to address earlier studies’ limitations by considering preventative and reduction interventions for physical punishment. By identifying facilitators and barriers, we hope to promote greater access to and implementation of these interventions, ultimately reducing physical punishment and creating a safer environment for children and youth globally.

## 2. Materials and Methods

**Figure 1:**
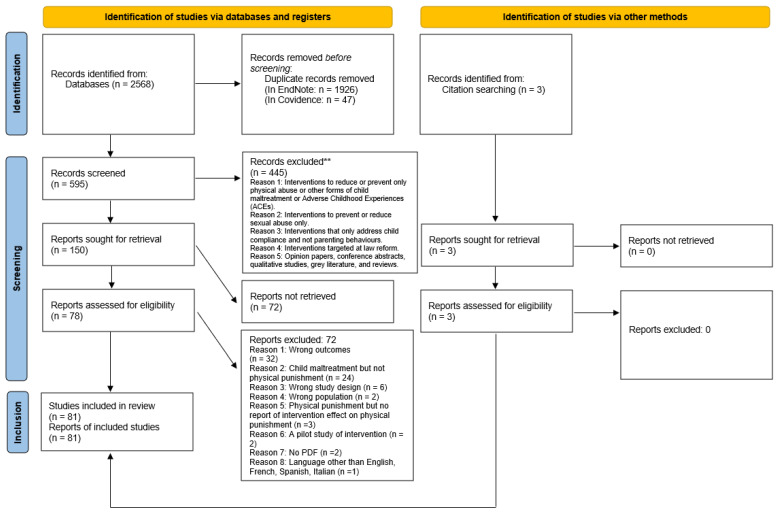
PRISMA-ScR flow diagram. ** IGD and AST screened all records. No automation tools were used to exclude studies.

A scoping review was conducted to address the research question and objectives, following the WHO Review Guide [54], the Joanna Briggs Institute 2020 Guide for Scoping Reviews [55], and the methodological work of Arksey and O’Malley [56] and Levac and Colquhoun [57]. A summary of the protocol was registered in the Open Science Framework “https://osf.io/d65ae/” (accessed on 30 October 2024). The search results and the study inclusion process are presented according to the Preferred Reporting Items for Systematic Reviews and Meta-analyses extension for scoping reviews in Figure 1 (PRISMA-ScR; Tricco, Lillie [58]). 

### 2.1. Search Strategy

The following online databases were searched: Scopus (*n* = 1048), Medline (*n* = 546), and Embase (*n* = 815) on OVID, as well as several EBSCOhost (*n* = 138) databases (Academic Search Complete, Family & Society Studies Worldwide, CINAHL, Child Development & Adolescent Studies, Criminal Justice Abstracts, Canadian Reference Centre, MasterFILE Premier, Social Work Abstracts, Women’s Studies International). The exact search terms are presented in Appendix A. Searches were created and carried out by an academic health sciences librarian (JL) with over 25 years of experience in knowledge synthesis (see Appendix A for more information on search strategy and search process).

### 2.2. Eligibility Criteria

Studies were included according to the Population, Concept, and Context framework. The definition of parenting intervention was kept as broad as possible to capture most parenting interventions. To this end, manualized, structured interventions targeting parenting behaviours and attitudes were included in this study. We did not specify the number of sessions, language, or inclusion of follow-up sessions as we wanted to capture most interventions. Parents and caregivers included biological parents, foster parents, family members and relatives caring for the children, and institutionalized parents. To be eligible for inclusion, studies had to (a) specify interventions and programs targeted at parents or caregivers to prevent and reduce physical punishment; spanking, slapping, and smacking; and physical or corporal punishment that may have included yelling and other emotional forms of punishment; (b) report on the delivery method; (c) report the setting of the intervention (e.g., one-to-one, group-based, app-based, and web-based interventions, as well as those in community centres and in health care settings); (d) mention if the parenting intervention/program was targeted at universal, selective, or indicated prevention; (e) be published between 2000 and 2022; (f) follow experimental and non-experimental study designs (to be able to include only quantitative studies); and (g) be in English, French, Italian, or Spanish (to comprehensively search for studies conducted in several geographical regions, and reflecting the team members fluency in each included language). Exclusion criteria are presented in Table 1. (See Appendix B for a summary of exclusion criteria).

### 2.3. Article Selection and Full-Text Review

Following the search, all identified citations were collated, uploaded, and deduplicated (see Figure 1). Before title and abstract screening, the team participated in rigorous training to ensure consistency among the reviewers. To guide the screening process, the reviewers were provided with a hierarchical title and abstract screening guide consisting of 5 questions that were first pilot tested and then refined with the team to ensure that the correct abstracts were being screened in and out. Two team members independently screened each title and abstract as ‘Yes’, ‘No’, or ‘Maybe’ based on the inclusion criteria. Inter-rater reliability between IGD and AST was calculated at the screening and full-text phases of the scoping review and measured by the Kappa coefficient. The inter-rater reliability between IGD and AST for title and abstract screening indicated good agreement (Kappa = 0.75). The inter-rater reliability between IGD and AST for full-text review indicated excellent agreement (Kappa = 0.91). AST or IDG decided on any disagreements between reviewers’ ratings.

Similarly, at the full-text article screening stage, the reviewers were provided with a hierarchical full-text review guide consisting of 4 questions. Each full-text article was independently reviewed by two reviewers and screened as either ‘Include’ or ‘Exclude’. If an article was excluded, the reviewer needed to indicate the reason for exclusion from a pre-determined list of reasons. If both reviewers selected to exclude a study at this phase, they were also required to indicate the same reason for exclusion before a study was removed. The final decision to include or exclude the paper, including the reason for exclusion, was determined through a discussion between AST and IDG, who reviewed the full text independently. This procedure ensured no relevant studies were being screened out unnecessarily. At the full-text review stage, relevant sources were retrieved, and their full texts were imported into Covidence (Version 2) scoping review software [59].

### 2.4. Data Extraction

Using the JBI Evidence Synthesis data extraction instrument [55], IGD and AST developed and pilot tested a modified data extraction tool for this study. The data extraction form was programmed into the Covidence 2.0 Data Extraction template. Before extracting data, the template was pilot tested by AST and IDG. This was an iterative process, and additional study variables were added in the early stages of the extraction process to allow for the most comprehensive data extraction process. The final data extraction form was divided based on the Population, Concept, and Context framework. It included sections on the characteristics of included studies (e.g., aim of study), information about parenting interventions (e.g., name of intervention, delivery and duration of intervention, instruments used in the study), and information on participants for each intervention (e.g., number of caregivers in the study, age, gender). Only one reviewer (IGD, JAMC, or SG) extracted data from each selected paper (see Data Extraction Categories in Appendix C). Six coding keys were created, in accordance with the Population, Concept, and Context framework (coding keys are available upon request).

### 2.5. Data Analysis

Frequencies and percentages were calculated in MS Excel (Version 2410) for each variable, according to the six coding keys.

## 3. Results

Upon the initial search of abstracts and titles, 2568 records were found. After a more detailed screening and de-duplication process, 595 citations were deemed eligible for further review. From this number of citations, 150 were sought for retrieval, and 78 reports were assessed for full-text eligibility. Additionally, three studies were identified through citation searching, sought for retrieval, and evaluated for eligibility. Finally, 81 articles were found to be eligible for full-text analysis, confirmed to be eligible, and included in this review (See Figure 1).

### 3.1. Objective 1: Identifying Evidence-Informed Interventions to Address Parental Use of Physical Punishment

#### 3.1.1. Characteristics of Included Studies

Descriptive information from each included study is presented in Table 2. The principal features of the studies examined in this review, including temporality and geographical representation, are described in Table 3. The publication history of the included studies indicates a general increase in the number of publications over time, from three (3.70%) papers published between 2000 and 2004 to 29 (35.80%) documents published between 2020 and 2022. Regarding the geographical location of the various studies, most studies relied on samples collected in North America (50.62%), followed by studies conducted with European samples (12.35%).

#### 3.1.2. Characteristics of Parent and Caregiver Participants

Data collected on parent/caregiver characteristics included the age and sex of the parents/caregivers examined and their relationship with the child (e.g., mother, father; see Table 4). Most studies included parents/caregivers across multiple age categories (42%); 32.10% included parents between 30 and 39 years old. It is important to mention that our results include the target population of parenting interventions, as was reported by the authors. Thus, most included studies reported male and female parents/caregivers (76.54%) attending the interventions, followed by studies targeting only female parents/caregivers (18.52%). Regarding the relationship between the parent/caregiver and the child, the results indicated that most interventions targeted mothers and fathers (53.09%).

#### 3.1.3. Characteristics of Children and Youth Participants

Data collected on children and youth age is presented in Table 5. The results indicate that most interventions targeted parents/caregivers with children and youth in multiple age ranges. Among those studies that reported child/youth data (82.72%), over 50% involved children in different age categories (58.02%). In contrast, fewer studies targeted parents/caregivers with younger children aged between one and four (14.81%).

#### 3.1.4. Sample Size and Study Design

The sample sizes for the included studies ranged from less than 50 participants to over 900 participants. Some studies reported sample sizes of 100 to 300 participants (35.80%), followed by fewer than 100 participants in their interventions (19.75%), and thirteen studies reported 300 to 500 participants (16.05%). The data indicated that most studies followed an experimental design corresponding to randomized controlled trials (RCT; 50.62%), followed by quasi-experimental studies without a control group (40.74%). Only a small number of studies were classified as either quasi-experimental with a control group (4.94%), observational (cohort and cross-sectional study; 3.70%), or other (experimental study with quasi-experimental components; 2.47%. See Appendix D for more information on sample size and study design).

#### 3.1.5. Parenting Interventions Reported in Included Studies

This scoping review indicated that most publications relied on manualized interventions (see Table 2). The most common programs were the ACT “Parent Raising Safe Kids” (8.64%), the Triple P Parenting Program (7.41%), and Parenting for Lifelong Health for Young Children (6.17%). Of note, the Positive Discipline in Everyday Parenting (PDEP) program was the only program implemented and studied in multiple countries [37,82] (3.70%).

#### 3.1.6. Transportation, Adaptation, and Contextualization of Parenting Interventions

This review also showed that many interventions were transposed, adapted, or contextualized (40.74%) before implementation. In contrast, the remaining studies either did not modify the intervention (29.63%) or did not report this information in the article (27.16%).

### 3.2. Objective 2: Assessing Reported Results of Parenting Interventions

The results of the included studies concerning parent and caregiver attitudes toward and/or use of physical punishment are presented in Table 6. Most studies (58.02%) reported decreased parental use of physical punishment. Other studies reported less endorsement of physical punishment following the completion of the intervention (27.16%). Four studies (4.94%) reported decreased use of physical punishment while also pointing out a reduction in anger expressions towards the child as salient intervention results.

### 3.3. Objective 3: Frequency, Setting, and Delivery Methods of Parenting Interventions and Children and Youth Reports on Intervention

Most interventions ranged from six to twelve sessions (50.62%). The settings of the interventions were categorized as home-visit-based, group-based, web-based/virtual, in-person, and multiple ways of delivery, as presented in Table 7. Studies were classified as in-person settings when the description mentioned that parents/caregivers attended the program in their respective settings and no additional information was provided for the mode of delivery. Most intervention settings were in person (43.21%), and nine interventions (11.11%) included multiple modes of delivery (in-person, web-based, video-based, home-visit-based, telephone-based, and videoconference-based). Four studies (4.49%) reported virtual (web-based and self-directed) interventions (See Table 8). Most interventions were conducted in English (37.04%) or Spanish (12.35%). This review also examined whether the included studies directly reported feedback from children and youth after the intervention. In most studies, children/youth feedback on intervention was generally not reported (87.65%).

### 3.4. Objective 4: Measures Used to Examine Behavioural and Attitudinal Changes Towards Physical Punishment

The present review also gathered data on the instruments used to assess the primary results of the included interventions (i.e., physical punishment; see Appendix E—Table A3). Researchers used various psychometrically established tools to evaluate parents’ use of physical punishment. The most common measures included the Adult Adolescent Parenting Inventory (AAPI-2; 14.81%), the Conflict Tactics Scale (CTS; 12%), and the Parenting Practices Interview (PPI; 7.41%). It is worth noting that although this study specifically examined physical punishment, most of the tools used in the included studies were composed of various forms of child maltreatment (e.g., physical punishment and emotional abuse), and the authors reported only on the findings related to physical punishment. More than half of the publications reviewed described the instruments’ reliability (e.g., Cronbach’s alpha, test–retest; 59.25%).

### 3.5. Objective 5: Identifying Facilitators and Barriers to Access, Use, and Implementation of Interventions

All the studies (81) included in this review commented on the strengths and limitations of using and implementing the respective interventions. Table 8 provides an overview of barriers and facilitators reported by authors in each study. Information on barriers and facilitators was reported by parents/caregiver participants, facilitators, and authors for each study. Educational resources to learn and understand child development and improve parenting practices were mentioned as a strength of the intervention in 30.28% of the studies examined, followed by positive child-rearing information as an alternative to physical punishment, mentioned in 25.69%. Notably, 8.26% of studies noted that culturally adapting the intervention to the target population facilitated access and implementation.

Concerning the barriers to access, use, and implementation of interventions, for those studies following a quasi-experimental design, limitations included the lack of a control group (11.76%) and that causal associations could not be determined from the study (2.94%)—changes in parental behaviours or attitudes post-test could not be attributed to the intervention. Some studies reported the lack of assessment of actual behaviour post-intervention (7.65%) as a limitation in assessing the effectiveness of interventions.

## 4. Discussion

This scoping review aimed to examine the nature of the available evidence on interventions to prevent and reduce parental use of physical punishment. To this end, we examined 81 peer-reviewed studies reporting on parenting programs or interventions to address physical punishment. All of the studies included in this review employed quantitative methodologies. Given that the methodological approach of a scoping review is inherently exploratory, we were particularly interested in synthesizing components of parenting interventions, such as the number of sessions, delivery mode, and setting. Thus, the inclusion of quantitative studies was deemed appropriate. However, we recognize the value of qualitative reviews in better understanding experiences and perceptions in serving universal, at-risk, and selective populations. A meta-synthesis approach to analyzing the extensive literature on parenting interventions could also yield valuable insights for improvement. The data extracted from the examined studies addressed the review’s research question and objectives by identifying evidence-informed interventions and the related components to address parental use of physical punishment.

### 4.1. Overview of Critical Findings

Concerning critical findings from this scoping review, most of the studies in this review were conducted in the United States and other Western countries. One notable exception included the studies on the PDEP program, implemented in 11 countries throughout Asia, Africa, the Middle East, South America, and Southeast Asia [38,82]. Based on these findings, additional research should focus on implementing and evaluating parenting interventions in LMICs. Efforts should be made in all areas (e.g., health, education, laws, social services) to advance the implementation of culturally respectful parenting interventions and regulations to eliminate physical punishment globally.

Results from the present scoping review indicated that only ten studies reported feedback from children and youth on the interventions; more research involving children and youth is needed. Children’s and youth’s views and perceptions on how parenting interventions are conducted and designed are essential to increasing the effectiveness of parenting programs and understanding the barriers and limitations to stopping violent intergenerational parenting practices. The importance of collaboration between government, not-for-profit agencies, researchers, practitioners, and end-users of studies (i.e., parents, caregivers, and children/youth) is highlighted as a strength toward using and implementing parenting interventions successfully [72].

Regarding the long-term impact of the interventions, several studies presented outcomes one year after the program’s completion, while another study reported outcomes at a seven-year follow-up. Benzies and colleagues [65] detailed a parenting intervention geared towards preschoolers and their parents, encompassing various facets of child development, including parenting and life skills education. The seven-year follow-up assessment indicated no significant decrease in attitudes toward physical punishment, parental distress, or empathy [65]. In contrast, Breitenstein and colleagues [67] reported on the Chicago Parent program, which aimed to help children aged 2–5 years and their parents by providing evidence-based strategies for building positive relationships and problem-solving skills. After one year, parents who underwent the program reduced their use of physical punishment compared to those who did not participate [67]. This underscores the importance of including educational components in interventions focused on problem-solving skills and child development. Similarly, Gross and colleagues [89] reported a decrease in the use of physical punishment at a 1-year follow-up among parents who participated in the Chicago Parent program.

### 4.2. Definition of Physical Punishment

Given the different definitions of child maltreatment and the inclusion of physical punishment as part of harsh parenting, one meta-analysis [52] provides an essential distinction between child maltreatment and harsh parenting. Harsh parenting includes ineffective and dysfunctional parenting, and it is often viewed as the start of child maltreatment. Harsh parenting is a negative parenting strategy that usually leads to severe consequences, such as physical injuries to the child [14].

The lack of consensus and the variations in definitions of physical punishment can lead to equivocal findings. For example, it hinders researchers from moving beyond the debate and considering ways to change cultural norms and policies regarding acceptable disciplinary practices [140]. Additionally, suppose parents are unaware that some disciplinary behaviours, such as spanking or slapping, are forms of physical punishment. [140]. In that case, they may be less likely to change their attitudes toward and use of this disciplinary strategy [140].

One systematic review comparing child maltreatment and harsh parenting instruments indicates essential considerations for future studies [141]. The authors highlight the importance of identifying specific parenting behaviours that differentiate between child maltreatment and harsh parenting [141]. They also emphasize the need for researchers to agree upon clear definitions and assessment methods for both child maltreatment and harsh parenting to improve future studies [141].

### 4.3. Key Components in Parenting Interventions to Address Physical Punishment

Important findings from this review indicate that the parenting intervention’s content (i.e., educational resources on child development and parenting practices) was a facilitator to influence parents in moving toward less endorsement and use of physical punishment after controlling for sociodemographic information, delivery method, and length of intervention (reported in 30% of studies). This finding suggests that future parenting programs should closely consider the content of the intervention alongside other factors (e.g., number of sessions, type of study design) when transporting or contextualizing interventions. Another important finding from this review indicates that only 8% of the studies reported that the intervention was adapted to follow culturally appropriate procedures. This aspect calls attention to the difficulty of contextualizing existing parenting programs and the need for research to understand best practices to guide implementation efforts for different populations to that which the intervention was developed and initially delivered in.

Regarding the barriers to access, use and implementation reported by authors, 20% of studies noted that parent self-report assessments were a barrier to adequately assessing the effectiveness of the intervention. It is essential to remember that changes in attitudes do not always equate to changes in behaviour. Parent self-report questionnaires and interviews should be combined with a multi-informant approach, including children and youth, as well as observational assessments from researchers or practitioners to provide a comprehensive view of the effectiveness of the intervention [142]. Most of the studies included in this review reported successfully reducing the practice of, and/or attitudes that support, parental use of physical punishment after the intervention; however, all of these used self-report assessments and may have been subject to social desirability bias.

It is recognized that some parent-reported tools used to measure physical punishment and child maltreatment lack responsiveness to assess changes over time (e.g., AAPI-2, CTSPC; [45]). For example, the AAPI-2 Value of Corporal Punishment subscale was insufficient in assessing responsiveness [47]. Some reasons for the lack of responsiveness include inconsistent results across studies, small sample sizes, and poor study quality; evidence supporting the included measures’ effectiveness was low [47]. It is imperative to utilize accurate measuring instruments aligned with the objectives of the intervention when assessing the efficacy of parenting interventions. Without reliable tools, the outcomes may be skewed, resulting in either the over- or under-reporting of the intervention’s effects.

It is crucial to note that integrating evidence-based criteria for assessing parenting interventions could improve our understanding of the efficacy of programs to reduce and prevent physical punishment. Bernedo and colleagues [143] highlighted several quality benchmarks for implementing family programs. The study by Bernedo and colleagues [143] does not explicitly address quality standards in interventions aimed at preventing and reducing the use of physical punishment. However, their evidence-based standards, as proposed, merit careful consideration. The authors underscore the significance of adhering to a theoretical model and delineating clear objectives as the primary pair of quality standards [143]. These aspects assume relevance within the context of this scoping review. By operating with a clear definition of physical punishment that encompasses explicit parental behaviours (e.g., slapping, spanking), the age bracket of the children (e.g., slapping a two-year-old as a punitive measure), and the purported rationale for the physical punishment (e.g., the intent to discipline the child or address parent–child discord), interventions can articulate their objectives and devise a coherent trajectory for implementation. One of the quality standards proposed by Bernedo and colleagues [143] involves incorporating the target recipients of interventions into the program implementation process. This ensures that their needs and preferences are considered throughout, which, in turn, can contribute to improved program outcomes.

### 4.4. Comparison of Results with Previous Reviews

The findings from the present study are similar to the results from a systematic review suggesting that parenting interventions are feasible and effective in improving parent-child relationships, parental knowledge of child development, and non-violent discipline practices in LMICs [50]. The findings from the present review are consistent with a narrative review of over 200 studies detailing evidence-based techniques to be used as alternatives for positive discipline [44]. Further, the narrative review by Quail and Ward [44] highlights the educational component of effective interventions and the need to test and review parent-child conflict de-escalation techniques to prevent harsh discipline. The results from this scoping review support this finding by suggesting that parenting interventions following an educational and psychoeducational component effectively reduce physical punishment in universal and at-risk parenting prevention efforts. Of note, the results of this scoping review also align with the findings of a systematic review by Santini and Williams [18] that stresses the need for RCTs and observational study designs when examining parenting interventions to ensure the change reported in parental behaviour is due to program components and attendance.

In a systematic review of parenting programs in LMICs, Knerr and colleagues [50] reported results favouring the intervention group on various parenting measures, including parent-child interaction, parent attitudes and knowledge, and reductions in harsh parenting. However, it is important to acknowledge that the adoption and adaptation of evidence-based interventions in low-resource settings may be influenced by different cultural beliefs about parenting and child behaviour, including family structures, language and literacy, poverty, social pressures, and practical issues such as a lack of water or electricity and safety concerns in areas of high violence [50,144]. Lachman and colleagues [98] and Katz and colleagues [92] highlight the value of considering contextual variables when designing interventions for at-risk parents in LMICs Interventions could foster positive changes in disciplinary practices by enhancing parenting practices, alleviating poverty, and improving access to prenatal and postnatal care, education, and economic support [92,98]. Taking environmental factors into account can further strengthen the effectiveness of these initiatives [98].

Evidence indicates that parenting interventions can effectively support families experiencing hardships, such as community violence or severe poverty [73,145,146]. A recent systematic review and meta-analysis has highlighted parenting interventions’ positive impact on reducing emotional and physical punishment in humanitarian contexts in LMICs [53]. These interventions are particularly relevant in challenging situations such as protracted conflicts, active warfare, refugee crises, disease outbreaks, and natural disasters [53]. The results demonstrate that these programs can effectively engage poor and vulnerable families, fostering substantial improvements in parenting practices, particularly in reducing physical punishment [53]. Additionally, these interventions enhance the mental health outcomes for children and parents, underscoring their value in promoting healthier family dynamics under challenging circumstances [53]. Making these interventions widely accessible is crucial, especially considering implementation factors [30,147]. Accessibility may be hindered due to disruptions in formal services that support families or address economic hardships [53,147,148].

This scoping review aimed to determine whether interventions were adapted and contextualized. Results similar to those presented by Knerr and colleagues [50] provide a more nuanced understanding of the factors to consider when adapting and contextualizing parenting programs to reduce violence against children. Other systematic reviews and meta-analyses have identified several effective components of parenting interventions, including short duration (up to 6 months), an intensity level based on family risk, parental self-confidence, and a combination of service delivery modes (e.g., home-based and clinic-based; Chen and Chan [52]). In contrast, a systematic review conducted by Gubbels and van der Put [49] highlighted the absence of significant moderating effects associated with contextual factors and structural elements, including duration, number of sessions, delivery location, and delivery setting.

### 4.5. Limitations

The findings of the present study must be considered in light of its limitations, which suggest areas for future research. First, we did not include studies that followed a qualitative methodology to explore parenting programs that aim to prevent or reduce physical punishment. It would be valuable to review qualitative studies on parenting interventions to understand how they work for specific populations, such as different age groups, ethnicities, and regions of residence. Qualitative studies would provide detailed information on parents’ experiences before, during, and after the intervention. Also, there is an increasing need to use mixed-method approaches to assess the effectiveness of parenting interventions, including qualitative reports of perceived experiences during the program. Second, we must carefully interpret our results as they may not apply to LMICs. While our review provides an overview of interventions aimed at preventing or reducing the use of physical punishment, it is limited by the published peer-reviewed evidence, primarily from Western countries. Therefore, the utility of this research is in presenting peer-reviewed and published studies from 2000 onwards in English to further the emergent implementation of parenting interventions to reduce physical punishment and guide future research and public policy efforts. Our results indicate that parenting interventions were undertaken primarily in Western countries, with a few exceptions (seven studies were conducted in South America [71,74,83,99,124,135,136], eight in Africa [73,97,111,113,125,134,139,147], and two were multi-site studies conducted in eleven countries [38,82]). Future research could benefit from including studies conducted worldwide to further our understanding of this emergent field.

### 4.6. Future Directions

Although beyond the scope of this study, potential future research may explore the impact of interventions on at-risk parents who report a decrease in the use of physical punishment after the program. Among the studies included in this scoping review, the research by Palusci and colleagues [116] focuses on the expectations and use of physical punishment among high-risk parents, such as those who are incarcerated, in residential substance abuse treatment facilities, receiving community referrals, or participating in community parenting programs, after undergoing a psychoeducation intervention (Family Nurturing Program). The results indicate that both male and female parents in all settings reported a significant increase in empathy and a decrease in the use of physical punishment after completing the program [116]. These findings suggest that interventions with a psychoeducational component effectively alter attitudes and behaviours related to physical punishment. Another study that examined the use of verbal and physical punishment among at-risk parents in low-income settings found a significant decrease in the use of these punishments at the one-month follow-up after the psychoeducational intervention [112]. These results underscore the importance of including child development education in interventions and providing alternative discipline techniques tailored to the child’s age.

There is a gap in the literature regarding successful recruitment methods for fathers in parenting interventions [131,149]. Future research could examine which recruitment techniques [124] are effective for including fathers in interventions. A recent study evaluated a parenting program (Program P) in Bolivia designed to actively engage men as caring fathers and partners [150]. The program aims to improve relationships among couples and between parents and their children while also working to prevent family violence. The findings indicate promising strategies for parenting interventions in LMICs and emphasize the importance of involving fathers in parenting initiatives [150]. This research opens up new avenues for supporting fathers and fostering healthier, more equitable relationships [150]. Specifically, the results from this study demonstrate the positive impact of home visits in actively engaging fathers and supporting their ongoing involvement [150]. These visits complemented group and individual sessions by emphasizing the importance of non-violent family relationships and addressing fathers’ aspirations [150].

Further, a qualitative formative research study conducted in Tanzania explored community perceptions of fatherhood, revealing valuable insights into the caregiving needs of fathers [151]. The findings underscore the potential benefits of creating culturally relevant, community-based initiatives that target fathers’ caregiving behaviours, strengthen marital relationships, and enhance psychosocial well-being [151]. By focusing on these areas, interventions can improve nurturing care and support for families in the community. These results contribute to a growing global body of evidence, particularly from Eastern and Southern Africa, demonstrating that the roles of fathers are evolving [151], increasingly moving beyond the traditional role of breadwinner to include more nurturing interactions with their young children [151,152,153]. The current evidence indicates that parenting interventions that include fathers are likely a cost-effective use of societal resources and can reduce the risk of physical punishment, child abuse, and neglect [154].

## 5. Conclusions

This scoping review offers a critical examination of the literature on parenting interventions that focus on reducing physical punishment against children and youth. With a universal prevention approach, the ultimate goal is to establish a culture of child safety, where institutions can implement simple, low-cost preventive programs that promote reporting and support and ultimately reduce the incidence of physical punishment across all populations. The results from this scoping review not only included studies focusing on addressing parenting behaviours and attitudes toward the use of physical violence but also assessed facilitators and barriers of access, use, and implementation of interventions, as reported by authors. The results of this scoping review could help guide the implementation and continued use of parenting interventions, considering delivery type, feedback from children and youth, and psychometric instruments used to measure change. Through this approach, we can foster a child safety culture that ensures every child’s well-being.

## Figures and Tables

**Table 1:** Exclusion criteria.

	Exclusion Criteria
Participants	Interventions targeted to reduce or prevent only physical abuse or other forms of child maltreatment or Adverse Childhood Experiences (ACEs) Interventions to prevent or reduce sexual abuse only Interventions including yelling/emotional abuse only Interventions that only address child compliance and not parenting behaviours
**Concept**	Interventions targeted to reduce or prevent only physical abuse or other forms of child maltreatment or ACEs Interventions targeted at law reform
**Context**	Opinion papers Conference abstracts Qualitative studies Grey literature (e.g., government reports, NGO reports) Case studies Systematic reviews, meta-analyses, network meta-analyses, narrative reviews, critical reviews, qualitative reviews

**Table 2:** Descriptive information on included studies.

	Author	Country/Region in Which the Study Was Conducted	Target Population	Parenting Program or Intervention
**1**	Abate, Marek [60]	United States	Indicated intervention	Triple P
**2**	Almeida, Abreu-Lima [61]	Portugal	Universal prevention	Multiple interventions
**3**	Álvarez, Rodrigo [62]	Spain	Universal prevention	Growing Up Happily in the Family (Creciendo Felices en la Familia).
**4**	Álvarez, Byrne [63]	Spain	Indicated intervention	Growing Up Happily in the Family (Creciendo Felices en la Familia).
**5**	Álvarez, Padilla [64]	Spain	Indicated intervention	Growing Up Happily in the Family (Creciendo Felices en la Familia).
**6**	Benzies, Mychasiuk [65]	Canada	Selective prevention	Two-Generation Preschool Programme
**7**	Bertero, Irons [66]	United States	Selective prevention	No Hit Zone (NHZ)
**8**	Breitenstein, Gross [67]	United States	Universal prevention	The Chicago Parent Program CPP
**9**	Byrne, Rodrigo [68]	Spain	Universal prevention	Apoyo Personal y Familiar (APF) Personal and Family Support program
**10**	Byrne, Salmela-Aro [69]	Spain	Indicated intervention	Personal and Family Support Program
**11**	Canfield, Weisleder [70]	United States	Selective prevention	Video interaction Project (VIP)
**12**	Cassiano Russo, Pizzarro Rebessi [71]	Brazil	Universal prevention	PROPAIS II
**13**	Chavis, Hudnut-Beumler [72]	United States	Selective prevention	Play Nicely
**14**	Cluver, Meinck [73]	South Africa	Universal prevention	Sinovuyo Caring Families Teen Programme
**15**	Cova, Rincón [74]	Chile	Universal prevention	Day by Day, Day by Day
**16**	Criss, Grant [75]	United States	Indicated intervention	Triple P
**17**	Demeusy, Handley [76]	United States	Indicated intervention	Building Healthy Children BHC
**18**	DePanfilis, Dubowitz [77]	United States	Indicated intervention	Family Connections FC.
**19**	De Zoysa, Siriwardhana [78]	Sri Lanka	Universal prevention	No name given only intervention program description
**20**	Duggan, McFarlane [79]	Hawaii	Indicated intervention	The Healthy Start Program model
**21**	Duggan, Caldera [80]	United States	Universal prevention	Healthy Families Alaska (HFAK)
**22**	Durrant, Plateau [81]	Canada	Universal prevention	Positive Discipline in Everyday Parenting PDEP
**23**	Durrant, Plateau [38]	Australia, Canada, Japan, Georgia, Kosovo, Palestine, Mongolia, Venezuela, Guatemala, Gambia, Paraguay, Philippines, Solomon Islands	Universal prevention	Positive Discipline in Everyday Parenting PDEP
**24**	Durrant, Stewart-Tufescu [82]	Australia, Japan, Philippines	Universal prevention	Positive Discipline in Everyday Parenting PDEP
**25**	Esteban, Mamani-Benito [83]	Peru	Universal prevention	ACT ‘Parents Raising Safe Kids’
**26**	Fayyad, Farah [84]	Lebanon	Selective prevention	Helping Challenging Children (Arabic adaptation)
**27**	Fergusson, Boden [85]	New Zealand	Universal prevention	Early Start
**28**	Gallitto, Romano [86]	Canada	Indicated intervention	SafeCare
**29**	Gonzalez, Ateah [87]	Canada	Universal prevention	Triple P
**30**	Grogan-Kaylor, Galano [88]	United States and Canada	Universal prevention	Mom’s Empowerment Program
**31**	Gross, Garvey [89]	United States	Universal prevention	The Chicago Parent Program CPP
**32**	Jansen, Frantz [90]	North Macedonia, Republic of Moldova, Romania	Universal prevention	Parenting for Lifelong Health for Young Children
**33**	Javier, Coffey [91]	United States	Universal prevention	Incredible Years Parenting Program
**34**	Katz, Jarrett [92]	United States	Selective prevention	Pride in Parenting
**35**	Kirkland, Lee [93]	United States	Indicated intervention	Healthy Families New York
**36**	Kleyn, Hewstone [94]	South Africa	Universal prevention	Parenting for Lifelong Health for Young Children
**37**	Knox, Burkhart [95]	United States	Indicated intervention	ACT ‘Parents Raising Safe Kids’
**38**	Knox and Burkhart [96]	United States	Indicated intervention	ACT ‘Parents Raising Safe Kids’
**39**	Lachman, Wamoyi [97]	Tanzania	Universal prevention	Skillful parenting and agribusiness programme
**40**	Lachman, Alampay [98]	Philippines	Universal prevention	Happy Family for Filipino Children
**41**	Lawrenz, Yousafzai [99]	Brazil	Selective prevention	ACT ‘Parents Raising Safe Kids’
**42**	LeCroy and Krysik [100]	United States	Universal prevention	Healthy Families Arizona
**43**	Leijten, Raaijmakers [101]	Netherlands	Selective prevention	Incredible Years Parenting Program
**44**	Letarte, Normandeau [102]	Canada	Indicated intervention	Incredible Years Parenting Program
**45**	Linares, Montalto [103]	United States	Indicated intervention	Incredible Years Parenting Program
**46**	Love et al., 2005 Love, Kisker [104]	United States	Universal prevention	Early Head Start
**47**	Magalhaes and Knox [105]	Portugal	Universal prevention	ACT ‘Parents Raising Safe Kids’
**48**	Maguire-Jack, Steinman [106]	United States	Indicated intervention	Triple P
**49**	McCoy, Lachman [107]	Thailand	Universal prevention	Parenting for Lifelong Health for Young Children
**50**	Miller, Weston [108]	United States	Indicated intervention	Parenting While Incarcerated
**51**	Minkovitz, Hughart [109]	United States	Universal prevention	The Healthy Steps for Young Children Program
**52**	Mitchell, Morawska [110]	Australia	Selective prevention	Triple P
**53**	Murphy, Embleton [111]	Kenya	Indicated intervention	Parenting for Lifelong Health for Young Children
**54**	Nicholson, Anderson [112]	United States	Indicated intervention	STAR Parenting Program
**55**	Ofoha and Saidu [113]	Nigeria	Universal prevention	Programas de Entrenamiento de Padres (PEP) Parenting Training Program
**56**	Oveisi, Ardabili [114]	Iran	Selective prevention	SOS! Help for Parents program
**57**	Ozyurt, Dinsever [115]	Turkey	Selective prevention	Triple P
**58**	Palusci, Crum [116]	United States	Indicated intervention	Helping Your Child Succeed (HYCS)
**59**	Pedro Solís-Cámara, Fox [117]	Mexico	Universal prevention	STAR Parenting Program
**60**	Porter and Howe [118]	United States	Indicated intervention	ACT ‘Parents Raising Safe Kids’
**61**	Portwood, Lambert [119]	United States	Selective prevention	ACT ‘Parents Raising Safe Kids’
**62**	Pruett, Cowan [120]	United States	Indicated intervention	Supporting Father Involvement (SFI)
**63**	Renzaho and Vignjevic [121]	Australia	Selective prevention	The African Migrant Parenting Program
**64**	Rerkswattavorn and Chanprasertpinyo [122]	Thailand	Universal prevention	No name given, only intervention program description
**65**	Richardson and Damashek [123]	Africa	Universal prevention	Play Nicely
**66**	Rincon, Cova [124]	Chile	Universal prevention	Day by Day, Day by Day
**67**	Robinson, Moss [125]	Africa	Universal prevention	Parenting for Child Development (P4CD)
**68**	Rothenberg, Lansford [126]	United States	Selective prevention	Fast Track
**69**	Sánchez-Cesáreo, Sánchez-Cardona [127]	Puerto Rico	Universal prevention	Parenting Fundamentals
**70**	Sangawi, Adams [128]	Iraq	Universal prevention	STEP programme
**71**	Scholer, Hamilton [129]	United States	Universal prevention	Play Nicely
**72**	Scholer, Hudnut-Beumler [130]	United States	Selective prevention	Play Nicely
**73**	Self-Brown, Osborne [131]	United States	Indicated intervention	Safe Care Dad to Kids Program Dad2K
**74**	Shaffer, Lindhiem [132]	United States	Selective prevention	The name of the intervention was not reported
**75**	Sim, Bowes [133]	Lebanon	Selective prevention	Families Make the Difference
**76**	Skar, Sherr [134]	Colombia	Universal prevention	International Child Development Programme (ICDP)
**77**	Skar, Sherr [135]	Africa	Selective prevention	International Child Development Programme (ICDP)
**78**	van der Kooij, Bipat [136]	Suriname	Universal prevention	Lobi Mi Pikin
**79**	Vives-Montero, Cortés-Pendón [137]	Spain	Universal prevention	Programas de Entrenamiento de Padres (PEP) Parenting Training Program
**80**	Waechter, Evans [138]	Grenada	Universal prevention	Conscious Discipline
**81**	Ward, Wessels [139]	South Africa	Universal prevention	Parenting for Lifelong Health for Young Children

**Table 3:** Sample characteristics.

	Count (%) *n* = 81
Year of publication	
2000–2004	3 (3.70)
2005–2009	7 (8.64)
2010–2014	23 (28.40)
2015–2019	19 (23.46)
2020–2022	29 (35.80)
Geographical region	
Africa	8 (9.88)
Asia	8 (9.88)
Australia, New Zealand	3 (3.70)
Europe	10 (12.35)
Middle East	1 (1.23)
Multiple Countries	2 (2.47)
North America	41 (50.62)
Polynesia	1 (1.23)
South America	7 (8.64)

**Table 4:** Characteristics of parent and caregiver participants.

	Count (%) *n* = 81
Parents/caregivers in intervention	
Age	
Multiple age categories	34 (42.0)
30–39	26 (32.10)
Not reported	9 (11.11)
20–29	7 (8.64)
40–49	5 (6.17)
Gender or sex	
Males and females	62 (76.54)
Females	15 (18.52)
Not reported	3 (3.70)
Males	1 (1.23)
Relationship to the index child	
Mothers and fathers	43 (53.09)
Mothers, fathers and extended family	19 (23.46)
Mother	15 (15.82)
Caregiver not specified	3 (3.70)
Father	1 (1.23)

**Table 5:** Characteristics of children and youth participants.

	Count (%) *n* = 81
Child/youth	
Age	
Multiple age categories	47 (58.02)
Not reported	14 (17.28)
1–4	12 (14.81)
5–9	6 (7.41)
<1	1 (1.23)
10–14	1 (1.23)

**Table 6:** Results of interventions to reduce physical punishment.

	Count (%) *n* = 81
Decrease in the use of physical punishment	47 (58.02)
Change in attitudes/less support towards physical punishment	22 (27.16)
No change in attitudes or use of physical punishment	8 (9.88)
Decrease in physical punishment and reduction in anger expressions towards the child	4 (4.94)

**Table 7:** Frequency, setting, and language of parenting interventions.

	Count (%) *n* = 81
Number of Sessions in the Intervention	
6 to 12	41 (50.62)
1 to 3	11 (13.58)
Not reported	11 (13.58)
21 to 40	8 (9.88)
13 to 20	5 (6.17)
More than 40	5 (6.17)
Setting of Intervention	
In-person	35 (43.21)
Home visit	17 (20.99)
Group-based	16 (19.75)
Multiple settings	9 (11.11)
Web-based/Virtual	4 (4.49)
Language of Intervention	
English	30 (37.04)
Not reported	26 (32.10)
Spanish	10 (12.35)
Other	4 (4.94)
Portuguese	3 (3.70)
Multiple languages (English, Spanish, Albanian, Arabic, Japanese, Mongolian)	3 (3.70)
Kurdish	1 (1.23)
French	1 (1.23)
Dutch	1 (1.23)
Japanese	1 (1.23)

**Table 8:** Facilitators and barriers to the use and implementation of identified interventions, as reported by authors.

	Count (%)
**Facilitators**	
Educational resources on child development, parenting practices	33 (30.28)
Positive child-rearing information (alternatives to physical punishment)	28 (25.69)
Culturally adapted intervention	9 (8.26)
Facilitators were members of the local community (staff/practitioners)	6 (5.50)
Monetary incentive/transportation	5 (4.59)
Potential to scale up intervention	5 (4.59)
Intervention effective in at-risk populations (court-mandated caregivers, prisons)	4 (3.67)
Cost-effective	4 (3.67)
The program was offered at multiple sites (home-based, family resource centres)	4 (3.67)
Partnerships with NGOs, government	3 (2.57)
Brief intervention (e.g., Play Nicely)	3 (2.75)
The program offered anger management	2 (1.83)
Standardized evaluation tools	1 (0.92)
Feedback from facilitators in role-play or discussion sections helped parents learn the content	1 (0.92)
Child reports of intervention and parent behaviour post-intervention	1 (0.92)
	**Count (%)**
**Barriers**	
Self-report assessments	34 (20.00)
Small sample size	28 (16.47)
No experimental design (lack of control group)	20 (11.76)
No assessment of actual behaviour post-intervention	13 (7.65)
Selection bias	12 (7.06)
Short follow-up	10 (5.88)
No follow-up	7 (4.12)
High attrition	7 (4.12)
Mostly or only mothers in the sample	7 (4.12)
Lack of validated measures	7 (4.12)
No information on motivation to attend the program	5 (2.94)
Causal associations cannot be determined from the study	5 (2.94)
Only cross-sectional data are available	5 (2.94)
Social desirability bias	5 (2.94)
Lack of baseline data	3 (1.76)

Note: Each study reported several facilitators and barriers.

## Appendix A

### Appendix A.1. Search Process, Search Strategies by Database, Review Software and, PRISMA Results

For this scoping review, it was essential to capture available studies with parenting interventions and physical punishment components. To this end, the librarian utilized a search strategy with three levels of increasing complexity. At the first level, the librarian used subject heading words such as ‘child abuse’ and ‘child maltreatment’ to define the overall field of the study. All relevant keywords like ‘physical punishment’ and ‘parenting interventions’ were identified at the second level, including their possible variations and synonyms. Finally, at the third level, all relevant keywords and index terms were identified, including their known variations, and Boolean operators were used to refine the search further. By carefully constructing the search strategy, the librarian ensured that all relevant studies were identified and included in the results. The search strategy yielded 2568 records from academic databases.

### Appendix A.2. Search Strategies by Database

MEDLINE

Ovid MEDLINE(R) and Epub Ahead of Print, In-Process, In-Data Review and Other Non-Indexed Citations and Daily <1946 to 19 August 2022>

1. Punishment/  6083
2. Child Abuse/  24,843
3. ((physical* adj4 disciplin*) or (physical* adj4 punish*) or spank* or corporal punishment).ti,ab.  1704
4. 1 or 2 or 3  31,252
5. Parenting/  20,146
6. (parent* or father* or mother*).mp.  769,660
7. 5 or 6  769,660
8. 4 and 7    8681
9. (prevent* or program* or intervention* or behavior* chang* or chang* behavior* or behaviour* chang* or chang* behaviour* or behavior* modif* or behaviour* modif* or modif* behaviour* or modif* behavior* or thrive or PPP or Triple P or positive parent* or healthy families).ti. 713,841
10. 8 and 9    813
11. limit 10 to (yr = “2000–Current” and (english or french)) 549

Embase

Embase <1974 to 2022 Week 32>

1. Child Abuse/   33,088
2. ((physical* adj4 disciplin*) or (physical* adj4 punish*) or spank* or corporal punishment).ti,ab.    1935
3. 1 or 2   34,522
4. Parenting/   57,390
5. (parent* or father* or mother*).mp.   964,578
6. 4 or 5    964,578
7. 3 and 6   10,190
8. (prevent* or program* or intervention* or behavior* chang* or chang* behavior* or behaviour* chang* or chang* behaviour* or behavior* modif* or behaviour* modif* or modif* behaviour* or modif* behavior* or thrive or PPP or Triple P or positive parent* or healthy families).ti.   867,030
9. 3 and 6 and 8   950
10. limit 9 to ((english or french) and (article or article in press or “review”))   811

Scopus

Scopus 21 August 2022 (*n* = 1048)

(TITLE-ABS-KEY(“physical punishment” OR “corporal punishment” OR spank* OR “physical* discipline*” OR “child abuse” OR “child maltreat*”) AND TITLE-ABS-KEY(parent* OR mother* OR father*) AND TITLE(PPP OR “triple p” OR prevent* OR program* OR intervention* OR “behaviour* change*” OR “behavior* change*” OR “behaviour* modif*” OR “behavior* modif*”)) AND (LIMIT-TO (PUBYEAR,2022) OR LIMIT-TO (PUBYEAR,2021) OR LIMIT-TO (PUBYEAR,2020) OR LIMIT-TO (PUBYEAR,2019) OR LIMIT-TO (PUBYEAR,2018) OR LIMIT-TO (PUBYEAR,2017) OR LIMIT-TO (PUBYEAR,2016) OR LIMIT-TO (PUBYEAR,2015) OR LIMIT-TO (PUBYEAR,2014) OR LIMIT-TO (PUBYEAR,2013) OR LIMIT-TO (PUBYEAR,2012) OR LIMIT-TO (PUBYEAR,2011) OR LIMIT-TO (PUBYEAR,2010) OR LIMIT-TO (PUBYEAR,2009) OR LIMIT-TO (PUBYEAR,2008) OR LIMIT-TO (PUBYEAR,2007) OR LIMIT-TO (PUBYEAR,2006) OR LIMIT-TO (PUBYEAR,2005) OR LIMIT-TO (PUBYEAR,2004) OR LIMIT-TO (PUBYEAR,2003) OR LIMIT-TO (PUBYEAR,2002) OR LIMIT-TO (PUBYEAR,2001) OR LIMIT-TO (PUBYEAR,2000)) AND (LIMIT-TO (DOCTYPE,”ar”) OR LIMIT-TO (DOCTYPE,”re”)) AND (LIMIT-TO (LANGUAGE,”English”) OR LIMIT-TO (LANGUAGE,”French”))

EBSCOhost

EBSCOhost 19 August 2022 (*n* = 138) Limits applied: Academic journals, 2000–current

Academic Search Complete, Family & Society Studies Worldwide, CINAHL, Child Development & Adolescent Studies, Criminal Justice Abstracts, Canadian Reference Centre, MasterFILE Premier, Social Work Abstracts, Women’s Studies International

(“physical punishment” OR “corporal punishment” OR spank* OR “physical* discipline*”) AND (Parent* OR mother* OR father* OR maternal OR paternal) AND TI (Prevent* OR program* OR intervention OR evidence OR “behavior* chang*” OR “chang* behavior*” OR “behaviour* chang*” OR “chang* behaviour*” OR “behavior* modif*” OR “behaviour* modif*” OR “modif* behavior*” OR “modif* behaviour*”)

### Appendix A.3. Review Software and PRISMA Results

The citations identified from the databases were uploaded into Covidence, a web-based data software program designed to streamline and systematize the production of high-quality systematic reviews [58]. The University of Manitoba provides an institution license to faculty and students to access the software. For this review, the 2568 citations identified from the databases were first imported into the reference manager software EndNote [155] and then uploaded into the project folder in Covidence, where citations were de-duplicated and removed from the review. The abstract and title screening, full-text article screening, and data extraction of included studies were conducted in Covidence. The final data extraction table and reference were exported into Excel as a CSV file.

### Appendix A.4. References

Babineau, J. (2014). Product review: Covidence (systematic review software). Journal of the Canadian Health Libraries Association/Journal de l’Association des bibliothèques de la santé du Canada, 35(2), 68–71 [59].

The EndNote Team. (2013). EndNote. In (Version EndNote 20) [64-bit]. Clarivate [155].

## Appendix B

### Exclusion Criteria

Studies were excluded if (a) the reported interventions targeted a reduction in or the prevention of physical abuse, yelling/emotional abuse only, or other forms of child maltreatment or Adverse Childhood Experiences (ACEs); (b) they included interventions that only addressed child compliance and not parenting behaviours; (c) they included interventions targeted at law reform; (d) they were opinion papers, conference abstracts, qualitative studies, grey literature, case studies, systematic reviews, meta-analyses, network meta-analyses, narrative reviews, critical reviews, or qualitative reviews. Eighty-one studies were eligible for full-text analysis.

## Appendix C

**Table A1:** Data extraction categories.

Study/Context Characteristics	Concept Characteristics: Design and Measurement	Concept Characteristics: Psychometric Instrument to Assess Physical Punishment	Population Characteristics
- Author and Year - Title - Funding sources - Study primary aim - Intervention primary results on physical punishment - Geographical region(s)	- Study design - Method of recruitment of participants - Name of program or intervention - Description of intervention: procedures, number of sessions, duration of intervention, follow-up, language of intervention, mode of delivery, participants in intervention, children and youth feedback on the intervention - Transporting, adapting, contextualizing intervention - Assessment of intervention	- Name of the instrument used to assess physical punishment as reported by parents and children/youth (if available) - Description of the instrument: items removed or adapted - Language of instrument - Validity and reliability of the instrument - Concordance measure between informants - Each study’s authors reported details about facilitators and barriers to implementing interventions.	- Sample size—number of parents/caregivers and children/youth in the intervention - Age of parent/caregiver - Sex of parent/caregiver - Parent/caregiver relationship to the index child - Sample size—number of children/youths in the intervention - Sex and age ranges of children/youth - Additional demographic information was extracted for the parent/caregiver and children/youth (if reported in studies): education level, income level, ethnicity, urban/rural/other location, and the number of children in the household at the time of intervention

## Appendix D

**Table A2:** Sample size and study design of included studies.

		Count (%) *n* = 81
Sample size		
	Less than 50	13 (16.05)
	Less than 100	16 (19.75)
	100 to 300	29 (35.80)
	300 to 500	13 (16.05)
	500 to 700	5 (6.17)
	700 to 900	3 (3.70)
	More than 900	2 (2.47)
	Less than 50	13 (16.05)
Study Design		
	Experimental study design (RCT)	41 (50.62)
	Quasi-experimental study with control group design	4 (4.94)
	Quasi-experimental study without control group design	33 (40.74)
	Observational study	3 (3.70)

## Appendix E

**Table A3:** Instruments used to report parent/caregiver’s use of physical punishment.

	Count (%) *n* = 81
**Instrument to measure physical punishment**	
Adult Adolescent Parenting Inventory (AAPI-2)	12 (14.81)
Alabama Parenting Questionnaire (APQ)	8 (9.88)
Attitudes Toward Spanking (ATS)	4 (4.94)
Child Behavior Checklist (CBCL)	1 (1.23)
Conflict Tactics Scale, Parent–Child Version (CTSPC); Conflict Tactics Scale (CTS)	10 (12.35)
Confusion, Hubbub, and Order Scale (CHAOS)	1 (1.23)
Dimensions of Discipline Inventory (DDI)	1 (1.23)
Discipline Module, Multiple Indicator Cluster Survey (MICS)	2 (2.47)
Humboldt State University (HSU) Parenting Survey	1 (1.23)
Instrument created for study	5 (6.17)
ISPCAN Child Abuse Screening Tool (ICAST-TRIAL).	5 (6.17)
Multiple instruments (APQ, Harsh Discipline Practice List—HDPL)	1 (1.23)
No instrument	3 (3.70)
No name provided	1 (1.23)
Parent Behaviour Checklist (PBC)	3 (3.70)
Parent Questionnaire	1 (1.23)
Parental Behavior Scale Short Version (PBS)	1 (1.23)
**Parental Discipline Behaviour Questionnaire (PDBQ)**	1 (1.23)
Parental Response to Child Misbehavior (PRCM)	1 (1.23)
Parenting Practices Interview (PPI)	6 (7.41)
Parenting Questionnaire (PQ)	1 (1.23)
Parenting Scale (PS)	4 (4.94)
Physical Punishment Beliefs Scale	1 (1.23)
Situational Questionnaire on Child-Rearing Practices; Situational questionnaire of child-rearing practices and goals	2 (2.47)
Socolar Discipline Survey	1 (1.23)
Standardized PDEP Pre and Post-Program Parent Questionnaires	1 (1.23)
The ACT Against Violence Parents Raising Safe Kids Scale	2 (2.47)
The Strengths and Difficulties Questionnaire (SDQ)	1 (1.23)
	**Count (%)** ***n* = 81**
**Validity and reliability of the instrument**	
Validity measures	1 (1.23)
Reliability measures	48 (59.25)
Validity and reliability measures	6 (7.40)
Not reported	26 (32.09)
